# Provision of small‐quantity lipid‐based nutrient supplements does not improve intestinal health among rural Malawian children

**DOI:** 10.1111/mcn.13331

**Published:** 2022-02-07

**Authors:** Zhifei Liu, Ulla Ashorn, Chilungamo Chingwanda, Kenneth Maleta, Lotta Hallamaa, Andrew Matchado, Emma Kortekangas, Kathryn G Dewey, Per Ashorn, Yue‐Mei Fan

**Affiliations:** ^1^ Center for Child, Adolescent and Maternal Health Research, Faculty of Medicine and Health Technology Tampere University Tampere Finland; ^2^ Department of Public Health University of Malawi Zomba Malawi; ^3^ Department of Nutrition University of California Davis CA USA; ^4^ Department of Paediatrics Tampere University Hospital Tampere Finland

**Keywords:** alpha‐1‐antitrypsin, calprotectin, children, intestinal health, nutrient supplements, regenerating 1B protein, rural Malawi

## Abstract

Lipid‐based nutrient supplements (LNS) have been found to improve child growth and reduce child mortality. However, the mechanistic pathways for these improvements warrant exploration. One potential pathway is linked to improvement in intestinal health. Our study aimed to test a hypothesis that small‐quantity LNS (SQ‐LNS) could reduce the levels of intestinal inflammation, repair and permeability of children. As intestinal health markers we measured fecal calprotectin, regenerating 1B protein (REG1B) and alpha‐1‐antitrypsin concentrations at 18 months of age (after 12 months of supplementation) and 1 year later (12 months after cessation of supplementation). In this analysis, we included data of 735 children who participated in a randomised dietary supplementation trial in rural Malawi; 243 children who received 20 g/day SQ‐LNS from 6 to 18 months of age were in the SQ‐LNS group, while the others who received no dietary supplementation during this period were in the control group. At 18 months of age, the mean concentrations of calprotectin, REG1B and alpha‐1‐antitrypsin were 241, 105 µg/g and 7.1 mg/dl, respectively, in the SQ‐LNS group, and 224, 105 µg/g and 7.4 mg/dl, respectively, in the control group, and did not differ between the SQ‐LNS and control groups. We conclude that SQ‐LNS provision did not have an impact on children's intestinal health in rural Malawi.

## Key messages

- Provision of small‐quantity lipid‐based nutrient supplements (SQ‐LNS) has proven useful in the promotion of children's growth but the mechanistic pathways are unclear.
- In a sample of rural Malawian children, provision of 20 g/day SQ‐LNS to 6‐ to 18‐month‐old children had no impact on concentrations of faecal calprotectin, REG1B, or alpha‐1‐antitrypsin at 18 months (which represented intestinal inflammation, repair and permeability).
- A 12‐month provision of SQ‐LNS, by itself, may not be sufficient to improve children's intestinal health in conditions like those of rural Malawi.

## Graphical abstract

Lipid‐based nutrient supplements (LNS) have been found to improve child growth and reduce child mortality. One potential pathway is linked to improvement in intestinal health. Our study in rural Malawi did not observe an effect of small‐quantity LNS on children's intestinal health.

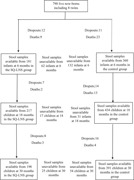

## 1 INTRODUCTION

Lipid‐based nutrient supplements (LNS) provided in large quantities have proven effective and are widely used in the treatment of children with severe acute malnutrition (Ciliberto et al., 2005; Hossain et al., 2020; Jadhav et al., 2019; Linneman et al., 2007). Recent evidence also suggests that provision of small‐quantity LNS (SQ‐LNS) to apparently nonmalnourished infants and young children can have a positive impact on their growth and development in some, but not all low‐income settings (Das et al., 2018, 2019; Dewey, Stewart, et al., 2021; Dewey, Wessells, et al., 2021; Prado et al., 2021). Moreover, the provision of SQ‐LNS is likely to reduce child mortality in low‐resource settings (Stewart et al., 2020). Given these positive impacts, the provision of SQ‐LNS is listed in a recent expert review as one of the key interventions to promote healthy child growth in low‐ and middle‐income countries (Keats et al., 2021).

Despite the promising results so far, the scale‐up of SQ‐LNS may be restricted by heterogeneity in impact on certain outcomes and the paucity of information about possible mechanistic pathways. The benefits of SQ‐LNS may come from improvements in intestinal microbiota diversity (Kamng'ona et al., 2020) which could lead to reduction in intestinal inflammation and damage, common in many low‐income settings (Luby et al., 2018). This improved intestinal health could increase nutrient absorption (Jumpertz et al., 2011) and facilitate the secretion of hormones that promote growth outcomes (Wong et al., 2016). Increased nutrient absorption could in turn lead to an improved intestinal immune response, which could further modify intestinal microbiota composition and reduce the risk of intestinal inflammation and permeability (Farre et al., 2020; Okumura & Takeda, 2017; Rodriguez et al., 2011; Shi et al., 2017). So far, however, only a few studies have focused on the impact of SQ‐LNS on intestinal health. One study in Bangladesh has reported that SQ‐LNS provided for 18 months between 6 and 24 months of age reduced intestinal inflammation at 14 months of age but increased intestinal inflammation and permeability at 28 months of age (Lin et al., 2020). Given these mixed results, further studies are needed to investigate the impact of SQ‐LNS on children's intestinal health.

In this study, we aimed to assess whether the provision of SQ‐LNS has an effect on intestinal health among rural Malawian children. We hypothesised that SQ‐LNS provided to children from 6 to 18 months of age would reduce faecal concentrations of calprotectin, REG1B and alpha‐1‐antitrypsin. Reduction in concentrations of these biomarkers served as indicator of reduced intestinal inflammation, reduced intestinal repair and reduced intestinal permeability, respectively (Costa et al., 2003; Guerrant et al., 2016; Kosek et al., 2013). To test this hypothesis, we compared concentrations of these biomarkers at the age of 18 months between children who had been either receiving or not receiving SQ‐LNS during the preceding 12 months. As a secondary analysis, to assess the persistence of any effect of SQ‐LNS, we compared concentrations of the same biomarkers in these children at 30 months of age, 12 months after the dietary supplementation had ceased.

## 2 METHODS

### 2.1 Study design and participants

This study is based on a randomised controlled trial, the international lipid‐based nutrient supplements DYAD (iLiNS‐DYAD) trial, conducted in Mangochi District, rural Malawi. Details of the trial and primary outcomes have been described elsewhere (Ashorn et al., 2015). In brief, a total of 1391 pregnant women were enroled and randomised to one of three groups: the SQ‐LNS group in which women received SQ‐LNS during pregnancy and 6 months thereafter, the iron and folic acid (IFA) group in which women received IFA during pregnancy, or the multiple micronutrients (MMN) group in which women received MMN during pregnancy and 6 months thereafter. There were 869 mothers who participated in a complete follow‐up. Their live‐born infants received dietary supplementation between 6 and 18 months of age and were followed up until the age of 30 months. This group formed the sampling frame for the current study. During the child supplementation period, mothers in the SQ‐LNS group received 140 g of SQ‐LNS weekly, with an instruction to provide 20 g of it daily to their study infants. Children born to women in the other groups received no dietary supplements.

In the present study, we analysed the impact of providing SQ‐LNS between 6 and 18 months of age on children's intestinal health. We considered intestinal health at 18 months of age as the key outcome for these analyses because the infants had not received any supplements before the age of 6 months and there were no effects of maternal supplementation on infant intestinal health biomarkers at 6 months of age. Thus, we combined children who received no supplementation (whose mothers were in the IFA or MMN groups) into one control group and compared their postintervention outcomes to children who did receive dietary supplementation between 6 and 18 months (whose mothers were in the SQ‐LNS group).

All pregnant women signed an informed consent form before joining the study and again before the child follow‐up. The study approval was obtained from ethics committees in Malawi (College of Medicine) and Finland (Pirkanmaa Hospital District).

### 2.2 Data collection

Study staff collected data on maternal age, household assets, drinking water source and sanitary facilities at enrollment with questionnaires and tested maternal HIV status using whole‐blood antibody rapid tests. They also recorded information on infant sex when infants were born, weight at 6 months using an electronic infant weighing scale (SECA 381 baby scale, Seca GmbH & Co.) and length at 6 months using the length board (Harpenden Infantometer, Holtain Limited). In addition, we calculated length‐for‐age z‐score (LAZ) and weight‐for‐length z‐score (WLZ) using World Health Organization Child Growth Standards (WHO Multicentre Growth Reference Study Group, 2006) and household assets z‐score based on building materials, sanitary facilities, lighting source, drinking water source and cooking fuel to represent socioeconomic status (Filmer & Pritchett, 2001).

### 2.3 Selection and measurement of biomarkers

We selected faecal calprotectin as an indicator of intestinal inflammation, faecal REG1B as a measure of intestinal repair and faecal alpha‐1‐antitrypsin as an indicator of intestinal permeability. These biomarkers were selected based on their demonstrated validity in trials and other studies on inflammatory bowel diseases and environmental enteric dysfunction (EED) in paediatric populations (R. K. Campbell et al., 2017; Naylor et al., 2015; Paduchova & Durackova, 2009).

We collected stool samples as previously described (Liu et al., 2020). In brief, study staff collected stool samples from children who were 6, 18 and 30 months old during home visits. They transported the samples to laboratories for aliquots in cryovial tubes and stored samples at −80℃ before shipping samples to a laboratory in Tampere University, Finland.

To quantify biomarker concentrations, we used commercial Dx faecal calprotectin ELISA kits (DH002; Hycult Biotech), REG1B ELISA kits (TECHLAB, Inc.) and human alpha‐1‐antitrypsin ELISA kits (PromoCell GmbH). These kits had demonstrated good sensitivity and specificity in samples from multiple paediatric trials (Asgarshirazi et al., 2017; Naylor et al., 2015; Peterson et al., 2013).

We thawed and diluted stool samples at 1:50 for calprotectin, 1:10000 for REG1B and 1:250 for alpha‐1‐antitrypsin and ran each plate of stool samples with controls and standards provided with the ELISA kits according to the procedures described by manufacturers. The lower detection limit in these assays for faecal calprotectin, REG1B and alpha‐1‐antitrypsin was 16, 6.25 µg/g and 1.8 mg/dl, respectively.

### 2.4 Statistical analysis

The statistical analysis was conducted with STATA 15.0 version (StataCorp). Measures of biomarker concentrations less than the lower limit of detection (LOD) for calprotectin, REG1B and alpha‐1‐antitrypsin were replaced, respectively, by 8, 3.13 µg/g and 0.9 mg/dl, which were half of the LOD for those respective biomarkers (European Food Safety Authority, 2010).

We calculated means and standard deviations for continuous variables and percentages for categorical variables. With a large sample size, parametric analysis of means is robust and valid regardless of a skewed distribution (Cheung, 2014). We used analysis of variance to test the statistical significance of any observed difference, with *p* < 0.05 indicating a statistically significant observation.

According to our preprepared statistical analysis plan (https://ucdavis.app.box.com/s/hk63i9b7zaaaksxxlxk8jayczwyykd7h), we conducted the main statistical analysis cross‐sectionally, from samples collected from 18‐month‐old children. At this age, when the children had received SQ‐LNS for 12 months, we hypothesised that there would be a difference in the mean faecal biomarker concentrations between the SQ‐LNS and control groups. However, given the theoretical possibility of a sustained effect of nutrient supplementation on microbiota associated with intestinal inflammation (Laursen et al., 2016), we did a secondary cross‐sectional analysis at the 30 months' time point.

In addition to unadjusted models, we analysed models adjusted for calprotectin, REG1B and alpha‐1‐antitrypsin at 6 months and models adjusted for the respective biomarker concentrations at 6 months of age plus infant sex, LAZ and WLZ at 6 months and other characteristics at enrollment including maternal age and HIV infection, household assets z‐score, drinking water source (piped water and borehole/wells, lake and river) and sanitary facilities (regular pit latrine and none/water closet and improved pit latrine). These covariates were selected because they had previously been associated with intestinal health of infants living in low‐income contexts and dietary intervention (D. I. Campbell et al., 2003; Gough et al., 2020; Keusch et al., 2014; Lin et al., 2020; Mullen et al., 2012)

In sensitivity analyses, we compared differences in means in log‐transformed concentrations of biomarkers between the SQ‐LNS and control groups at 18 and 30 months of age. In addition, we performed unadjusted analyses to compare mean concentrations of respective biomarkers among the three intervention groups at 18 and 30 months of age.

## 3 RESULTS

**Figure 1:**
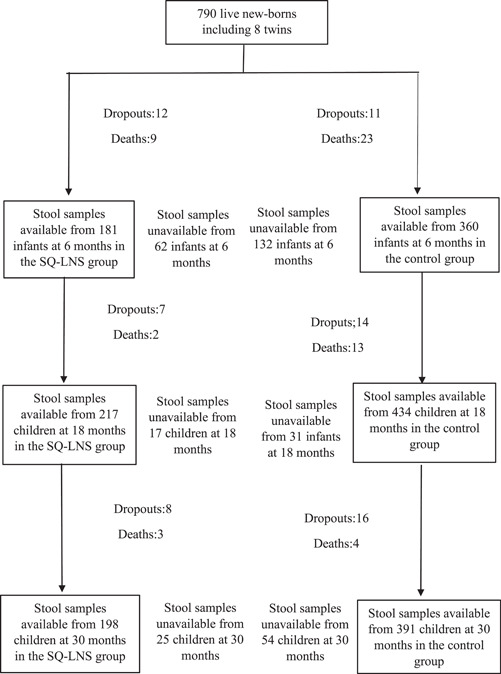
Flow chart of participants

Out of 790 live‐born infants, 32 died and 23 dropped out before the age of 6 months, leaving 735 children at 6 months in the study. Stool samples were available for 541 children at 6 months, 651 at 18 months and 589 at 30 months (Figure 1).

At 6 months of age, the mean (SD) LAZ and WLZ of the participants was −1.3 (1.1) and 0.4 (1.1), respectively, and 32% were stunted and 2% wasted. The mean faecal concentration of calprotectin, REG1B and alpha‐1‐antitrypsin was 555, 193 µg/g and 29.7 mg/dl, respectively. The SQ‐LNS and control groups were similar in terms of these characteristics as well as maternal HIV status and age and socioeconomic characteristics including drinking water and sanitary facilities (Table 1). There were 55 children (7%) who died, dropped out, or provided no stool samples and hence were excluded from all analyses: 21 in the SQ‐LNS group and 34 in the control group. The excluded and included children had similar mean characteristics at 6 months (Table S1).

**Table 1:** Characteristics of the study participants by intervention group^a^

Characteristic	SQ‐LNS group (*N* = 243)	Control group (*N* = 492)
Proportion of boys	49% (118)	47% (230)
LAZ at 6 months	−1.3 (1.1)	−1.3 (1.2)
Proportion of stunted children at 6 months (LAZ < −2)	35% (52)	31% (103)
WLZ at 6 months	0.4 (1.1)	0.4 (1.2)
Proportion of wasted children at 6 months (WLZ < −2)	3% (6)	2% (9)
Calprotectin concentration (µg/g) at 6 months	559 (558)	553 (527)
REG1B concentration (µg/g) at 6 months	203 (191)	188 (155)
Alpha‐1‐antitrypsin concentration (mg/dl) at 6 months	35.3 (107.3)	26.9 (76.2)
Proportion with maternal HIV	12% (29)	12% (59)
Age of mothers, years	25.1 (6.2)	25.1 (5.8)
Household assets z‐score	0.0 (1.0)	−0.1 (1.0)
Drinking water source, piped water or borehole	88% (215)	89% (434)
Sanitary facilities, regular pit latrine or none	90% (219)	90% (441)

Abbreviations: HIV, human immunodeficiency virus; LAZ, length‐for‐age z‐score; REG1B, regenerating 1B protein; SD, standard deviation; SQ‐LNS, small‐quantity lipid‐based nutrient supplements; WLZ, weight‐for‐length z‐score.

^a^ Values were mean (SD) or percentages and none of the variables in this table differed between the SQ‐LNS and control groups.

At 18 months, the proportion of stool samples that had a biomarker concentration below the LOD was 2% for calprotectin, 24% for REG1B and 15% for alpha‐1‐antitrypsin. The mean concentrations of calprotectin, REG1B and alpha‐1‐antitrypsin were 230, 105 µg/g and 7.3 mg/dl, respectively. No significant differences in these biomarkers were observed between the SQ‐LNS and control groups. Adjustment of the analyses for respective biomarker concentrations at 6 months or and other selected potential confounders did not change the results (Table 2).

**Table 2:** Concentration of intestinal biomarkers in SQ‐LNS versus control groups at age 18 months^a^

	Mean (SD)		Difference in means (95% CI)					
	SQ‐LNS group (*N* = 217)	Control group (*N* = 434)	Model 1^b^	*p* value	Model 2^c^	*p* value	Model 3^d^	*p* value
Calprotectin, µg/g	241 (338)	224 (347)	−17 (−73, 39)	0.551	−12 (−79, 56)	0.728	4 (‐66, 74)	0.915
REG1B, µg/g	105 (138)	105 (141)	1 (−23, 24)	0.952	−2 (−28, 24)	0.877	−11 (−38, 16)	0.417
Alpha‐1‐antitrypsin, mg/dl	7.1 (9.1)	7.4 (17.8)	0.3 (−2.3, 2.9)	0.828	1.0 (−2.7, 4.6)	0.600	1.4 (−2.7, 5.4)	0.505

Abbreviations: CI, confidence interval; REG1B, regenerating 1 B protein; SD, standard deviation; SQ‐LNS, small‐quantity lipid‐based nutrient supplements.

^a^ Values were mean (SD).

^b^ Model 1 was unadjusted analysis.

^c^ Model 2 was adjusted for calprotectin, REG1B and alpha‐1‐antirypsin concentration at 6 months respectively.

^d^ Model 3 was adjusted for calprotectin, REG1B and alpha‐1‐antirypsin concentration at 6 months, child sex, LAZ and WLZ at 6 months, maternal HIV status (yes/no) and age and household assets z‐score, drinking water source (piped water and borehole/wells, lake and river) and sanitary facilities (regular pit latrine and none/water closet and improved pit latrine). All *p* values and differences in means were obtained from the analysis of variance.

At 30 months, the mean concentrations of these three biomarkers decreased to 150, 58 µg/g and 3.5 mg/dl, respectively. There were no significant differences between the SQ‐LNS and control groups in either unadjusted or adjusted models (Table 3).

**Table 3:** Concentration of intestinal biomarkers in SQ‐LNS versus control groups at age 30 months^a^

	Mean (SD)		Difference in means (95% CI)					
	SQ‐LNS group (*N* = 198)	Control group (*N* = 391)	Model 1^b^	*p* value	Model 2^c^	*p* value	Model 3^d^	*p* value
Calprotectin, µg/g	137 (210)	157 (382)	20 (−37, 78)	0.485	18 (−46, 83)	0.573	25 (−45, 94)	0.484
REG1B, µg/g	56 (98)	59 (107)	3 (−15, 21)	0.707	4 (−18, 26)	0.702	5 (−18, 29)	0.655
Alpha‐1‐antitrypsin, mg/dl	3.5 (3.4)	3.5 (6.8)	−0.0 (−1.0, 1.0)	0.972	0.2 (−1.3, 1.6)	0.801	0.5 (−1.1, 2.0)	0.563

Abbreviations: CI, confidence interval; REG1B, regenerating 1 B protein; SD, standard deviation; SQ‐LNS, small‐quantity lipid‐based nutrient supplements.

^a^ Values were mean (SD).

^b^ Model 1 was unadjusted analysis.

^c^ Model 2 was adjusted for calprotectin, REG1B and alpha‐1‐antirypsin concentration at 6 months, respectively.

^d^ Model 3 was adjusted for calprotectin, REG1B and alpha‐1‐antirypsin concentration at 6 months, child sex, LAZ and WLZ at 6 months, maternal HIV status (yes/no) and age and household assets z‐score, drinking water source (piped water and borehole/wells, lake and river) and sanitary facilities (regular pit latrine and none/water closet and improved pit latrine). All *p* values and differences in means were obtained from analysis of variance.

A sensitivity analysis using log‐transformed values confirmed that there were no intergroup differences in these biomarker concentrations at 18 months (Table S2) or 30 months (Table S3).

Another sensitivity analysis, in which children whose mothers had received IFA or MMN were analysed separately, showed similar results as the main analysis, both at 18 months (Table S4) and at 30 months (Table S5).

## 4 DISCUSSION

This study aimed to assess the impact of providing children with SQ‐LNS for 12 months, from 6 to 18 months of age, on intestinal health, as assessed by faecal calprotectin, REG1B and alpha‐1‐antitrypsin. In a sample of 735 rural Malawian children with available data, daily provision of SQ‐LNS for 12 months was not associated with reductions in the degree of intestinal inflammation, repair or permeability in 18‐month‐old‐children. We also did not observe differences in concentrations of these intestinal biomarkers at 30 months of age between the children who had previously received SQ‐LNS and those who had not.

The validity of our findings may theoretically have been affected by procedures for faecal sample collection and storage, incomplete blinding of participants to the trial intervention (LNS vs. no LNS), loss to follow‐up, combining the IFA and MMN groups in the analysis, skewed distribution of biomarker concentrations and a limited panel of biomarkers. To minimise the possibility of bias, we used standardised sample collection, processing, storage and analysis procedures that, for example, ensured timely freezing of samples after collection. The outcome variables were not subjectively assessed, and laboratory personnel and data managers were blinded to the intervention until the database was frozen. Only 7% of the participants were lost to follow‐up, giving credibility to the sample findings (Schulz & Grimes, 2002). Results from the main analysis in which children in the IFA and MMN groups were combined into one comparison group were similar to those from the sensitivity analysis in which the three randomised groups were compared. In addition, the use of log‐transformed biomarker concentrations did not change the results. Finally, the selected biomarkers of intestinal inflammation, repair and increased permeability covered most of the features that are considered typical for environmental enteropathy (Gough et al., 2020). Therefore, we believe that our sample findings are valid and indicate that in this target group, LNS supplementation did not improve children's intestinal health.

Our finding of no effect of SQ‐LNS on REG1B is consistent with results from a trial performed in Bangladesh, but the results for alpha‐1‐antitrypsin are different (Lin et al., 2020). These two studies used SQ‐LNS with a similar composition, provided the same daily dose and included a similar age range for the sample populations. The study in Bangladesh found that SQ‐LNS provided for 18 months, between 6 and 24 months of age, had no impact on the concentration of alpha‐1‐antitrypsin at 14 months of age (8 months after the intervention was initiated) but increased it at 28 months of age (4 months after the intervention ceased). The authors speculated that the increased faecal concentration of alpha‐1‐antitrypsin after the intervention might reflect an SQ‐LNS‐induced shift in the timing of the deterioration in intestinal inflammation that typically occurs among young children in such environments (Lin et al., 2020). In our Malawian target group, however, we did not observe a similar deterioration or shift in its timing, as the mean concentration of alpha‐1‐antitrypsin was lower at 30 months than at 18 months of age and similar in the SQ‐LNS and control groups at both time points.

The impact of SQ‐LNS provision on the intestinal health of 6‐ to 18‐month‐old infants has also been studied in the SHINE trial in Zimbabwe. In this factorial design trial, infants received either standard care, water and sanitation‐related intervention package (WASH) from birth to 18 months of age, infant and young child feeding (IYCF) support and 20 g of SQ‐LNS from 6 to 18 months or both WASH and IYCF (Sanitation Hygiene Infant Nutrition Efficacy Trial et al., 2015). Similar to our study, there were no differences in the mean faecal REG1B, alpha‐1‐antitrypsin or other EED biomarker concentrations between children who did and did not receive SQ‐LNS, at any age between 6 and 18 months (Gough et al., 2020). The mean concentrations of both faecal REG1B and alpha‐1‐antitrypsin at 18 months were higher in Zimbabwean children than in our Malawian sample.

Possible explanations for the lack of impact of SQ‐LNS on intestinal health in our study are as follows. First, although a 6‐ to 12‐month long SQ‐LNS supplementation, starting from the age of 6 months, has been associated with growth and mortality benefits (Das et al., 2019; Dewey, Wessells, et al., 2021; Stewart et al., 2020), it has not markedly altered intestinal microbiota composition which is one pathway by which intestinal inflammation could have been altered (Cheung et al., 2016; Kamng'ona et al., 2020). Second, the concentrations of the biomarkers of intestinal inflammation and permeability that we used are typically high in infancy and decrease during the first 2 years of life (McCormick et al., 2017; van Elburg et al., 2003), which is consistent with our observation of rapid declines in the mean concentrations of three biomarkers between 6 and 30 months of age. This physiological decrease in these biomarkers, as well as the role of breastfeeding in reducing intestinal inflammation (Miyake et al., 2020), may limit the ability to detect the effects of nutritional intervention. Third, the increased child mobility and hand‐mouth behaviours during the age interval for this intervention could lead to high pathogen exposure (Morita et al., 2017), making it less likely that SQ‐LNS, by itself, would be sufficient to improve intestinal health.

In summary, our data do not support the hypothesis that providing SQ‐LNS to infants from 6 to 18 months of age would reduce the levels of intestinal inflammation, repair and permeability among children in rural Malawi. Further research including additional indicators of intestinal health, and in other paediatric populations, is needed to understand the effects of SQ‐LNS on these outcomes.

## CONFLICT OF INTERESTS

The authors declare no conflict of interests.

## AUTHOR CONTRIBUTIONS

ZL, UA, LH, PA and Y‐MF designed the study; all authors conducted the research; ZL performed statistical analysis; LH and PA advised on the statistical analysis; ZL drafted the manuscript; all authors reviewed and commented the draft manuscript; all authors reviewed and approved the final manuscript for submission.

## Supporting information

Supporting information.

## Data Availability

The data will be available from the authors upon reasonable request.
