# Supplementary material for: Provision of small‐quantity lipid‐based nutrient supplements does not improve intestinal health among rural Malawian children
Source: Matern Child Nutr. 2022 Feb 7;18(3):e13331. doi: 10.1111/mcn.13331 (PMC9218311; doi:10.1111/mcn.13331)
Supplement: Supplementary file 1 — Supporting information. [file MCN-18-e13331-s001.docx]

Supplemental Table 1 Characteristics of the study participants included and excluded at 6 months^†^

| Characteristic | Included  N=735 | Excluded  N=55 | *P*-value |
| --- | --- | --- | --- |
| Proportion of boys | 47% (348) | 52% (24) | 0.546 |
| Proportion with maternal HIV | 12% (88) | 13% (6) | 0.822 |
| Age of mothers, years | 25.1 (5.9) | 24.8 (7.2) | 0.808 |
| Household assets z-score | -0.0 (1.0) | 0.3 (1.0) | 0.053 |
| Drinking water source, piped water or borehole | 89% (649) | 90% (46) | 1.000 |
| Sanitary facilities, regular pit latrine or none | 90% (660) | 96% (50) | 0.219 |

^†^Values were mean (SD) or percentages.

Abbreviations: HIV, human immunodeficiency virus; SD, standard deviation.

## Supplemental Table 2 Log-transformed concentrations of intestinal biomarkers in SQ-LNS versus control groups at age 18 months †

|  | Mean (SD) | | Difference in means (95% CI) | | | | | |
| --- | --- | --- | --- | --- | --- | --- | --- | --- |
|  | SQ-LNS group (N=217) | Control group (N=434) | Model1^a^ | *P*-value | Model2^b^ | *P*-value | Model3^c^ | *P*-value |
| Log (calprotectin) | 2.1 (0.5) | 2.1 (0.5) | -0.0 (-0.1, 0.1) | 0.509 | 0.0 (-0.1, 0.1) | 0.999 | 0.0 (-0.1, 0.1) | 0.798 |
| Log (REG1B) | - 1. (0.8) | 1.5 (0.8) | 0.0 (-0.1, 0.1) | 0.913 | 0.0 (-0.1,0.2) | 0.774 | 0.0 (-0.1, 0.2) | 0.908 |
| Log (alpha-1-antitrypsin) | 0.7 (0.4) | 0.6 (0.4) | -0.0 (-0.1, 0.1) | 0.885 | 0.0 (-0.1, 0.1) | 0.693 | 0.0 (-0.1, 0.1) | 0.480 |

^†^Values were mean (SD). ^a^ Model 1was unadjusted analysis. ^b^ Model 2 was adjusted for calprotectin, REG1B and alpha-1-antirypsin concentration at 6 months respectively. ^c^ Model 3 was adjusted for calprotectin, REG1B and alpha-1-antirypsin concentration at 6 months, child sex, LAZ and WLZ at 6 months, maternal HIV status (yes/no) and age, and household assets z-score, drinking water source (piped water and borehole / wells, lake, and river), and sanitary facilities (regular pit latrine and none / water closet and improved pit latrine). All p values and difference in means were obtained from analysis of variance.

Abbreviations: CI, confidence interval; REG1B, regenerating 1 B protein; SD, standard deviation; SQ-LNS, small-quantity lipid-based nutrient supplements.

## Supplemental Table 3 Log-transformed concentrations of intestinal biomarkers in SQ-LNS versus control groups at age 30 months ^†^

|  | Mean (SD) | | Difference in means (95% CI) | | | | | |
| --- | --- | --- | --- | --- | --- | --- | --- | --- |
|  | SQ-LNS group (N=198) | Control group (N=391) | Model1^a^ | *P*-value | Model2^b^ | *P*-value | Model3^c^ | *P*-value |
| Log (calprotectin) | 1.8 (0.5) | 1.8 (0.5) | -0.0 (-0.1, 0.1) | 0.781 | -0.0 (-0.1 0.1) | 0.882 | -0.0 (-0.1, 0.1) | 0.866 |
| Log (REG1B) | 1.2 (0.7) | 1.2 (0.7) | 0.0 (-0.1, 0.2) | 0.587 | 0.0 (-0.1, 0.2) | 0.800 | 0.0 (-0.1, 0.2) | 0.546 |
| Log (alpha-1-antitrypsin) | 0.4 (0.4) | 0.4 (0.4) | -0.0 (-0.1, 0.0) | 0.532 | 0.0 (-0.1, 0.1) | 0.941 | 0.0 (-0.1, 0.1) | 0.495 |

^†^Values were mean (SD). ^a^ Model 1was unadjusted analysis. ^b^ Model 2 was adjusted for calprotectin, REG1B and alpha-1-antirypsin concentration at 6 months respectively. ^c^ Model 3 was adjusted for calprotectin, REG1B and alpha-1-antirypsin concentration at 6 months, child sex, LAZ and WLZ at 6 months, maternal HIV status (yes/no) and age, household assets z-score, drinking water source (piped water and borehole/wells, lake, and river), and sanitary facilities (regular pit latrine and none/water closet and improved pit latrine). All p values and difference in means were obtained from analysis of variance.

Abbreviations: CI, confidence interval; REG1B, regenerating 1 B protein; SD, standard deviation; SQ-LNS, small-quantity lipid-based nutrient supplements.

## Supplemental Table 4 Concentration of intestinal biomarkers between three intervention groups at age 18 months ^†^

|  | Mean (SD) | | | Global *P*-value^a^ | Difference in means (95% CI) | | | | | |
| --- | --- | --- | --- | --- | --- | --- | --- | --- | --- | --- |
|  | SQ-LNS group (N=217) | IFA group (N=218) | MMN group (N=216) |  | SQ-LNS and IFA group | *P*-value^b^ | SQ-LNS and MMN group | *P*-value^b^ | IFA and MMN group | *P*-value^b^ |
| Calprotectin, µg/g | 241 (338) | 220 (286) | 228 (400) | 0.817 | 21 (-44, 86) | 0.531 | 13 (-52, 78) | 0.686 | 7 (-58, 72) | 0.825 |
| REG1B, µg/g | 105 (138) | 107 (138) | 104 (144) | 0.975 | -2 (-29, 25) | 0.873 | 1 (-26, 28) | 0.954 | -3 (-30, 24) | 0.828 |
| Alpha-1-antitrypsin, mg/dl | 7.1(9.1) | 7.1 (18.0) | 7.7 (17.6) | 0.913 | -0.0 (-3.0, 3.0) | 0.994 | -0.6 (-3.6, 2.4) | 0.710 | 0.6 (-2.4, 3.5) | 0.712 |

^†^Values were mean (SD). ^a^*P* value was obtained from analysis of variance. ^b^*P* value was obtained from multiple comparisons without adjustment.

Abbreviations: CI, confidence interval; IFA, iron-folic acid supplements; MMN, multiple micronutrients supplements; REG1B, regenerating 1 B protein; SD, standard deviation; SQ-LNS, small-quantity lipid-based nutrient supplements.

## Supplemental Table 5 Concentration of intestinal biomarkers between three intervention groups at age 30 months^†^

|  | Mean (SD) | | | Global *P*-value^a^ | Difference in means (95% CI) | | | | | |
| --- | --- | --- | --- | --- | --- | --- | --- | --- | --- | --- |
|  | SQ-LNS group ((N=198) | IFA group (N=206) | MMN group (N=185) |  | SQ-LNS and IFA group | *P*-value^b^ | SQ-LNS and MMN group | *P*-value^b^ | IFA and MMN group | *P*-value^b^ |
| Calprotectin, µg/g | 137(210) | 160 (433) | 154 (317) | 0.774 | -23 (-88, 42) | 0.490 | -17 (-85, 50) | 0.610 | -6 (-72, 61) | 0.871 |
| REG1B^§^, µg/g | 56 (98) | 53 (88) | 66 (125) | 0.462 | 3 (-18, 23) | 0.810 | -10 (-31, 11) | 0.349 | 13 (-8, 33) | 0.237 |
| Alpha-1-antitrypsin, mg/dl | 3.5 (3.4) | 3.6 (9.0) | 3.3 (3.1) | 0.931 | -0.1(-1.3,1.1) | 0.879 | 0.1(-1.1,1.3) | 0.821 | -0.2 (-1.4,1.0) | 0.706 |

^†^Values were mean (SD). ^a^*P* value was obtained from analysis of variance. ^b^*P* value was obtained from multiple comparisons without adjustment.

Abbreviations: CI, confidence interval; IFA, iron-folic acid supplements; MMN, multiple micronutrients supplements; REG1B, regenerating 1 B protein; SD, standard deviation; SQ-LNS, small-quantity lipid-based nutrient supplements.
